# Evolved resistance to a novel cationic peptide antibiotic requires high mutation supply

**DOI:** 10.1093/emph/eoac022

**Published:** 2022-05-30

**Authors:** Alfonso Santos-Lopez, Melissa J Fritz, Jeffrey B Lombardo, Ansen H P Burr, Victoria A Heinrich, Christopher W Marshall, Vaughn S Cooper

**Affiliations:** Department of Microbiology and Molecular Genetics, and Center for Evolutionary Biology and Medicine, University of Pittsburgh, Pittsburgh, PA, 15219 USA; Present address: Department of Microbiology, Hospital Universitario Ramon y Cajal, Instituto Ramón y Cajal de Investigación Sanitaria (IRYCIS), Madrid, Spain; Present address: Department of Microbial Biology, National Center of Biotechnology (CNB), Madrid, Spain; Department of Microbiology and Molecular Genetics, and Center for Evolutionary Biology and Medicine, University of Pittsburgh, Pittsburgh, PA, 15219 USA; Department of Microbiology and Molecular Genetics, and Center for Evolutionary Biology and Medicine, University of Pittsburgh, Pittsburgh, PA, 15219 USA; Department of Microbiology and Molecular Genetics, and Center for Evolutionary Biology and Medicine, University of Pittsburgh, Pittsburgh, PA, 15219 USA; Department of Microbiology and Molecular Genetics, and Center for Evolutionary Biology and Medicine, University of Pittsburgh, Pittsburgh, PA, 15219 USA; Department of Microbiology and Molecular Genetics, and Center for Evolutionary Biology and Medicine, University of Pittsburgh, Pittsburgh, PA, 15219 USA; Center for Evolutionary Biology and Medicine, University of Pittsburgh, Pittsburgh, PA, USA; Present address: Department of Biological Sciences, Marquette University, Milwaukee, WI, USA; Department of Microbiology and Molecular Genetics, and Center for Evolutionary Biology and Medicine, University of Pittsburgh, Pittsburgh, PA, 15219 USA; Center for Evolutionary Biology and Medicine, University of Pittsburgh, Pittsburgh, PA, USA

**Keywords:** antimicrobial resistance, mutation rate, *Pseudomonas aeruginosa*, cationic peptide, *Acinetobacter baumannii*

## Abstract

**Background and Objectives:**

A key strategy for resolving the antibiotic resistance crisis is the development of new drugs with antimicrobial properties. The engineered cationic antimicrobial peptide WLBU2 (also known as PLG0206) is a promising broad-spectrum antimicrobial compound that has completed Phase I clinical studies. It has activity against Gram-negative and Gram-positive bacteria including infections associated with biofilm. No definitive mechanisms of resistance to WLBU2 have been identified.

**Methodology:**

Here, we used experimental evolution under different levels of mutation supply and whole genome sequencing (WGS) to detect the genetic pathways and probable mechanisms of resistance to this peptide. We propagated populations of wild-type and hypermutator *Pseudomonas aeruginosa* in the presence of WLBU2 and performed WGS of evolved populations and clones.

**Results:**

Populations that survived WLBU2 treatment acquired a minimum of two mutations, making the acquisition of resistance more difficult than for most antibiotics, which can be tolerated by mutation of a single target. Major targets of resistance to WLBU2 included the *orfN* and *pmrB* genes, previously described to confer resistance to other cationic peptides. More surprisingly, mutations that increase aggregation such as the *wsp* pathway were also selected despite the ability of WLBU2 to kill cells growing in a biofilm.

**Conclusions and implications:**

The results show how experimental evolution and WGS can identify genetic targets and actions of new antimicrobial compounds and predict pathways to resistance of new antibiotics in clinical practice.

## INTRODUCTION

The ability of a bacterial population to evolve resistance to an antibiotic depends on several factors including the availability of mutations that increase resistance and the strength of selection imposed by the compound. Experimental evolution coupled with whole genome sequencing (WGS) is a powerful strategy to characterize genetic mechanisms of resistance. Propagation of a bacterial population in the presence of an antibiotic will eventually select those clones that are capable of surviving the antibiotic exposure, and WGS of these populations or clones will reveal genetic causes of the resistance phenotype. This method is especially powerful when studying cationic peptides, as multiple mutations may be needed to evolve resistance to them [1–3].

WLBU2 (also called PLG0206) is an engineered amphipathic alpha helix derived from the LL-37 peptide that inserts into the bacterial membrane and leads to cell death. WLBU2 is shown to be highly effective against ESKAPE (***E****nterococcus faecium*, ***S****taphylococcus aureus*, ***K****lebsiella pneumoniae*, ***A****cinetobacter baumannii*, ***P****seudomonas aeruginosa* and ***E****nterobacter spp.*) pathogens *in vitro* and *in vivo* [4–6]. Antibiotic activity is typically measured against planktonic cells as biofilms are highly antibiotic tolerant, but WLBU2 has demonstrated a high activity against *P. aeruginosa* and *S. aureus* biofilms [7, 8].

Despite challenging several Gram-negative and Gram-positive pathogens with WLBU2 *in vitro* and in animal models, only *P. aeruginosa* has been observed to develop resistance to WLBU2 after long periods of exposure [4]. However, the mechanism of resistance remains undefined. Identifying the genes and the subsequent mutations that confer resistance to novel antibiotics is crucial as it enables more comprehensive understanding of the modes of action of this new antimicrobial compound and how resistance to them evolves. Furthermore, these discoveries can increase our ability to predict the emergence of antimicrobial resistance (AMR) in clinical scenarios [9–11]. We hypothesized that resistance to WLBU2 is rare because of its effectiveness and because it requires a specific combination of mutations. Therefore, a large mutation supply may be needed to generate the narrow range of combinations of resistance determinants. A large mutation supply could be generated by an increase in mutation rate, large population sizes and/or long periods of time [12]. Due to the effectiveness of WLBU2 at killing and keeping population sizes small, we anticipated that increased mutation rates or prolonged subinhibitory exposure would be the most likely means for the bacteria to generate a larger mutation supply. We tested each of these strategies using experimental evolution coupled with genomic sequencing to define mechanisms of resistance to this new cationic peptide.

## METHODS

### Experimental evolution

We performed three independent evolution experiments. In all of them, we propagated the ancestor strains in a rich medium modified from M9 (referred to as M9^+^) containing 0.37 mM CaCl_2_, 8.7 mM MgSO_4_, 42.2 mM Na_2_HPO_4_, 22 mM KH2PO_4_, 21.7 mM NaCl, 18.7 mM NH_4_Cl and 0.2 g/l glucose and supplemented with 20 ml/l MEM essential amino acids (Gibco 11130051), 10 ml/l MEM nonessential amino acids (Gibco 11140050) and 10 ml each of trace mineral solutions A, B and C (Corning 25021-3Cl).

In the first experiment, we selected a single clone of *A. baumannii* ATCC17978 and *P. aeruginosa* PA14 and propagated them independently for 24 h in M9^+^ in the absence of antibiotic. We then sub-cultured each population into 10 replicate populations. Ten of the populations (five planktonic and five biofilm, methods described below) were propagated every 24 h in increasing concentrations of WLBU2 starting at 0.5× the minimum inhibitory concentration (MIC). We doubled the WLBU2 concentrations after 72 h until no population survived the treatment. As a control, we propagated the same number of strains both planktonically and in biofilm in the absence of antibiotic. To test the general ability of the experimental design for selecting populations resistant to cationic peptides, we also propagated populations of *A. baumannii* in each lifestyle with increasing concentrations of polymyxin B. All planktonic populations, and four out of five biofilm populations, survived in four times the MIC of polymyxin B, mainly through mutations in the *pmrABC* operon and in genes modifying lipid A (Supplementary Table S1).

In the second experiment, we propagated five planktonic populations and five biofilm populations under the same conditions described above, but instead using a hypermutator strain of *P. aeruginosa* obtained from Flynn *et al*. [13]. We also propagated three planktonic populations and three biofilm populations of the hypermutator strain in the absence of antibiotic pressure.

In the third experiment, we propagated five planktonic populations of the non-hypermutator laboratory strain *P. aeruginosa* PA14 under subinhibitory concentrations of WLBU2 for 10 days. Then, we propagated the populations that survived the treatment with 1× MIC of WLBU2 for 2 days to evaluate stability of the resistance phenotype.

All evolution experiments were performed using 18 mm glass tubes (Supplementary Fig. S1). For the planktonic propagation, we serially passaged 50 µl into 5 ml of M9^+^ (dilution factor 100), which corresponds to ∼6.64 generations per day. For biofilm populations, we transferred a polystyrene bead (Cospheric, Santa Barbara, CA, USA) to fresh media containing three sterile beads. We rinsed each bead in PBS before the transfer, therefore reducing the transfer of planktonic cells. Each day, we alternated between black and white marked beads, ensuring that the bacteria were growing on the bead for 24 h, which corresponds to ∼6–7.5 generations/day [14].

In all three experiments, we froze 1 ml of the surviving populations at Days 1, 3, 4, 6, 7, 9, 10 and 12 in 9% of DMSO (before and after increases in antibiotics).

### Phenotypic characterization: antimicrobial susceptibility and aggregation assay

We determined the MIC to WLBU2 and polymyxin B of the whole populations and four clones by broth microdilution according to the Clinical and Laboratory Standards Institute guidelines (CLSI 2019), in which each bacterial sample was tested in 2-fold increasing concentrations of each antibiotic. WLBU2 was provided by Peptilogics (Pittsburgh, PA, USA) and polymyxin B was provided by Alfa Aesar (Ward Hill, MA, USA). We performed this experiment with three replicates in the of antibiotic. Statistical comparisons of MIC values used log_2_ transformed values. Differences in the grand means between populations were evaluated by one-way ANOVA using the aov function from the stats package in R followed by the *post-hoc* multiple comparisons Dunnett’s test using the glht function.

To determine the aggregation ability of the four clones selected in the subinhibitory experiment, we grew two replicates (R1 and R2) of each clone and the ancestral strain in 5 ml of M9+ for 24 h at 37°C in a roller drum at 200 RPM. After 24 h, we transferred the whole culture of each clone to a 13 mm glass tube, and let the tubes settle over 24 h at 4°C without shaking. We vortexed replicate 1 (R1) of each clone for 30 s and measured the OD_600_. From R2, we carefully took 200 μl of the upper fraction without vortexing and measured the OD_600_. The aggregation percentage was estimated as 100(1–OD_600_ R2/1–OD_600_ R1). We performed this experiment with five replicates both in the absence and presence (4 μg/ml) of WLBU2. We compared the mean aggregation percentages of each clone by two-way ANOVA. *Post-hoc* pairwise comparisons between clones were conducted with the cld function of the multcomp package with the Šidák correction for multiple comparisons.

### Genome sequencing

We performed whole population genome sequencing of population that survived the treatment in each experiment as well as control lines with a coverage of 148.01 ± 42.80. This includes three biofilm and two planktonic populations of hypermutator populations surviving WLBU2 treatment and four biofilm and three planktonic populations evolved in the absence of the antibiotic (12 total populations). In addition, we sequenced the two populations and four clones that survived the subinhibitory WLBU2 treatment, as well as one untreated control population. Finally, we sequenced three biofilm and three planktonic populations of *A. baumannii* that were propagated in presence of polymyxin B as well as three planktonic and three biofilm populations propagated without polymyxin B (12 total populations).

We revived each population or clone from a freezer stock in the growth conditions under which they were isolated and grew for 24 h. DNA was extracted using the Qiagen DNAeasy Blood and Tissue kit (Qiagen, Hilden, Germany). The sequencing library was prepared as described by Turner *et al*. [14] according to the protocol of Baym *et al.* [15], using the Illumina Nextera kit (Illumina Inc., San Diego, CA, USA) and sequenced using an Illumina NextSeq500.

### Data processing

Sequences were filtered for quality and trimmed using Trimmomatic v0.36 [16] with the criteria: LEADING:20 TRAILING:20 SLIDINGWINDOW:4:20 MINLEN:70. We used breseq v0.31.0 [17] to call variants using the default parameters and the -p flag when identifying polymorphisms. The version of *A. baumannii* ATCC 17978-mff (GCF_001077675.1 downloaded from the NCBI RefSeq database, 17-Mar-2017) was used as the reference genome for variant calling. We added the two additional plasmid sequences present in the *A. baumannii* strain (NC009083, NC_009084) to the chromosome NZ_CP012004 and plasmid NZ_CP012005. The version of *P. aeruginosa* UCBPP-PA14 was downloaded from RefSeq on 25 August 2020. To remove probable false positives, we excluded mutations if they never reached a cumulative frequency of 25% across multiple time points or if also found in the ancestor’s genome. Additionally, to distinguish between broth-adaptive and WLBU2-adaptive mutations, we subtracted mutations found in the populations evolved in the absence of WLBU2. The *mutS* clone used in this study is a merodiploid bearing both the ancestral and the mutated *mutS* gene [13]. As breseq can fail to detect mutations when analyzing repeated sequences [17], we manually visualized the *mutS* locus within the BAM files, confirming that the mutation was present in the ancestral clone and in all evolved populations sequenced at *ca.* 50% frequency.

## RESULTS AND DISCUSSION

### Failure to evolve resistance using laboratory strains and standard protocols

**Figure 1.**
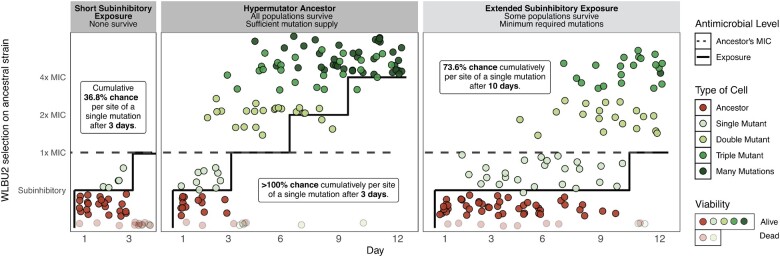
Schematic overview of the experimental design. We exposed wild-type *P. aeruginosa* PA14 (WT) to three different treatments of WLBU2. In the first one (left panel), we grew populations in subinhibitory concentrations of WLBU2 for 3 days, and then doubled this concentration to inhibitory concentrations that caused extinction. In the second experiment (middle panel), we used a hypermutator PA14 strain with a 116-fold greater mutation rate than WT. In the third one (right panel), we propagated WT for 10 days in subinhibitory concentrations of WLBU2, thus increasing the mutation supply by augmenting the number of generations. Dots simulate the expected number of mutants as calculated in Table 1

To identify mechanisms of resistance to WLBU2, we propagated the laboratory strains of *A. baumannii* ATCC 17978 and *P. aeruginosa* PA14 that had no prior exposure to WLBU2. We followed a protocol successfully used previously with several pathogens and different antibiotics, in which large populations of bacteria are treated with increasing concentrations (Fig. 1) [18–21]. This protocol selects for bacteria with increased resistance measures and sufficient growth compared to the ancestor by either increasing tolerance or resistance, but for simplicity, we refer to populations that survived the treatment as ‘resistant’ strains. We serially passaged both strains (bottleneck of ∼10^7^ cfu) in WBLU2 concentrations initially at half of the MIC and increasing concentration 2-fold every 3 days, up to four times the MIC. We propagated the same number of populations in the absence of WLBU2 to distinguish between adaptation to the environment and WLBU2-related mutations. Knowing that biofilm formation can influence the evolution of resistance [18, 20], we implemented this regimen in both planktonic and biofilm cultures (see Supplementary Fig. S1, http://evolvingstem.org and [18, 20] for details of the biofilm propagation). No populations survived exposure to the MIC of WLBU2. This suggested that there may be a narrow genetic pathway to gain WBLU2 resistance that could require more than one mutation.

The two main factors affecting the likelihood for a microbial population to evolve antibiotic resistance are: (i) the mutation supply that is determined by the product of the population size and mutation rate and (ii) the strength of selection imposed by the antibiotic [22]. If we assume a conservative mutation rate of 0.001 mutations per genome per cell division, and a Poisson distribution of the mutations in the genome [23], this experiment only had a cumulative probability of *∼*0.35 for *A. baumannii* and 0.29 for *P. aeruginosa* that any given nucleotide had been mutated at least once (in one cell) following 3 days of growth (∼36 generations) in subinhibitory concentrations, i.e. before being exposed to lethal concentrations (Table 1 and Supplementary Table S2 for detailed calculations). It is therefore possible that the correct mutation or combination of mutations needed to survive the antibiotic treatment had not occurred, or had been lost from the transfer bottleneck, before facing inhibitory concentrations. We therefore pursued two approaches to increase the number of mutations sampled in our *P. aeruginosa* populations to cultivate resistance to WLBU2: increasing the mutation rate and extending the duration of subinhibitory exposure (Fig. 1).

**Table 1.** Estimated mutation supply and distribution of mutations in the three different experiments

	Previously used protocol	Adapted protocols	
		Hypermutator ancestor	Extended subinhibitory exposure
Strain (*Pseudomonas aeruginosa*)	PA14	PA14 *mutS*: T114P	PA14
Mutation rate (mutation/genome/division)^a^	0.001	0.1	0.001
Length of exposure (days)^b^	3	3	10
Total cell divisions^c^	2.98 × 10^9^	2.98 × 10^9^	9.91 × 10^9^
Total number of mutations^d^	2.98 × 10^6^	2.98 × 10^8^	9.91 × 10^6^
Mean mutations per nucleotide, cumulative^e^	0.458	53.18	1.52
Cumulative probability of a mutation per nucleotide^f^	36.8%	99.6%	73.6%
**Results**			
Populations that survived entire experiment (%)	0%	50%	40%

a Calculated in Harris *et al*. [25].

b Days propagated before facing inhibitory concentrations of WLBU2.

c Calculated as the sum of cell divisions to regrow the population each day following dilution into fresh culture.

d Total cell divisions multiplied by mutation rate.

e Total mutations divided by chromosome size, 6.5 × 10^6^ pb.

f Based on Poisson distribution of the mutations. For details of the calculations, see Supplementary Table S2.

### Increasing mutation supply by using a hypermutator strain promotes evolution of resistance

**Figure 2.**
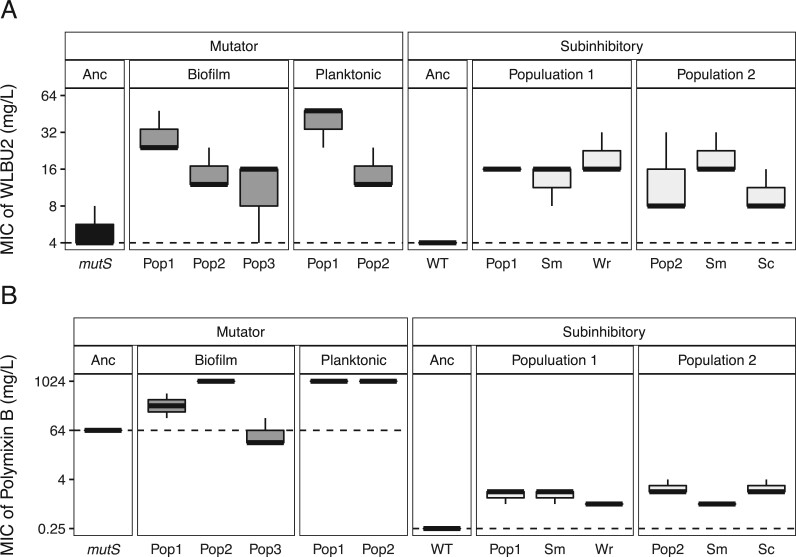
Resistance levels to WLBU2 and polymyxin B of populations evolved in the presence of WLBU2, as minimum inhibitory concentration (MIC) (mg/l). Boxplots show the median and quartiles for three replicates. Populations evolved from the *mutS* hypermutator genotype are in dark gray and resistant clones isolated from the ancestor are in light gray. All populations significantly exceed the WT resistance to WLBU2 and polymyxin B except biofilm Population 3 from the *mutS* ancestor. Data were analyzed by one-way ANOVA corrected for multiple comparisons using Dunnett’s *post hoc* test (*P* < 0.05). Dotted line denotes the MIC of the ancestor.

One way to increase the mutation supply during evolution experiments is to use an ancestral strain with a higher mutation rate. Hypermutator strains facilitate the evolution of resistance even to combination therapies owing to the increase in mutation rate [21]. These hypermutator genotypes also commonly evolve during chronic infections of *P. aeruginosa* and are therefore relevant to AMR evolution [24]. We selected a strain of *P. aeruginosa* PA14 with a defect in mismatch repair (*mutS* T112P), which has a mutation rate 116 times higher than the ancestor [13, 25] and a starting level of resistance to WLBU2 of 5.3 ± 2.3 mg/l (Fig. 2A). With this hypermutator strain, under the same experimental conditions described above and summing all individual cell divisions, each nucleotide experiences ∼>50 mutations over the first 3 days of growth under subinhibitory conditions (Table 1). Five hypermutator populations were propagated in each lifestyle combination following the previously described protocol. Two planktonic and three biofilm populations survived this treatment, resulting in 3–7.5-fold increases in resistance level relative to the ancestor (Fig. 2A). The other five populations did not survive when exposed to the inhibitory concentration the MIC of WLBU2.

We sequenced genomes from all five surviving populations to a depth of 148.0 ± 42.80 and detected a total of 98 mutations at frequencies > 0.1, including 31 fixed mutations (Supplementary Table S3). As the ancestor was a hypermutator strain, it can be difficult to infer what mutations were the drivers of the resistance phenotype and which were hitchhikers [26]. We focused on instances of gene-level parallel (repeated) evolution as strong candidates because mutations in the same gene found in independently derived lineages provide strong evidence of selection on this trait. Furthermore, the large population size and high mutation supply empowers selection to enrich the most beneficial genotypes [20, 27, 28].

Only four genes, *orfN*, *pmrB*, *wspF* and *morA*, were mutated in more than one population exposed to WLBU2 and not in the populations evolving in the absence of antibiotics, indicating roles for these mutations in evading the antibiotic treatment (Supplementary Tables S2 and S3). The two-component regulator *pmrB* governs several modifications of lipopolysaccharides (LPS) and has previously been demonstrated to confer resistance to other cationic peptides [29]. Four of the five WLBU2-resistant populations also show cross-resistance to polymyxin B (Fig. 2B) increasing their MIC from 2 to ≥4 folds. The *wspF* gene encodes a methylesterase that regulates activity of the surface-sensing Wsp cluster that in turn activates the diguanylate cyclase WspR and biofilm production [30, 31]. The *morA* gene encodes multiple sensor domains that control diguanylate cyclase and phosphodiesterase domains acting on the second messenger cyclic di-GMP. This molecule promotes biofilm production at high levels and motility at low levels [30, 32]. These biofilm-associated mutations in *wspF* and *morA* strongly indicate that production of aggregates or biofilm plays a role in resisting WLBU2.

### Extending the exposure to subinhibitory concentrations

**Figure 3.**
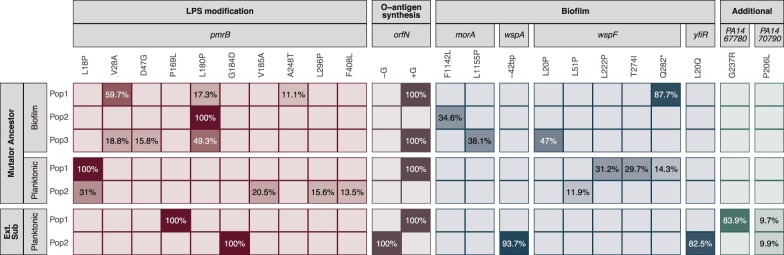
Whole-genome sequencing of the populations reveals parallel mutations in a few genes reaching high frequencies in all populations, suggesting that a minimum of two mutations among three key functional categories is required to increase resistance to WLBU2. While most fixed mutations were seen in the hypermutator-derived populations, genetic hitchhiking is more likely in these conditions. We therefore focused on parallel, high-frequency mutations seen in both the extended subinhibitory (Ext. Sub.) phase and hypermutator-founded experiments (indicative of adaptive targets and driver mutation). A complete list of mutations is shown in Supplementary Tables S3 and S4. More details of parallel mutations are shown in Supplementary Table S5

The hypermutator genotype of *P. aeruginosa* facilitated the evolution of resistance to WLBU2 (Fig. 2A), but isolating clones without other background mutations was impossible because of their increased mutation rate. Nonetheless, the parallelism in *orfN*, *morA*, *wspF* and *pmrB* (Fig. 3) provides a strong indicator of the fitness benefits of these mutations [28, 33]. Because resistance could evolve by increasing the mutation supply, we tested whether increasing the number of generations in subinhibitory concentrations of WLBU2 would increase the chance for the WT (non-hypermutator) ancestor of acquiring mutations needed to survive inhibitory concentrations.

**Figure 4.**
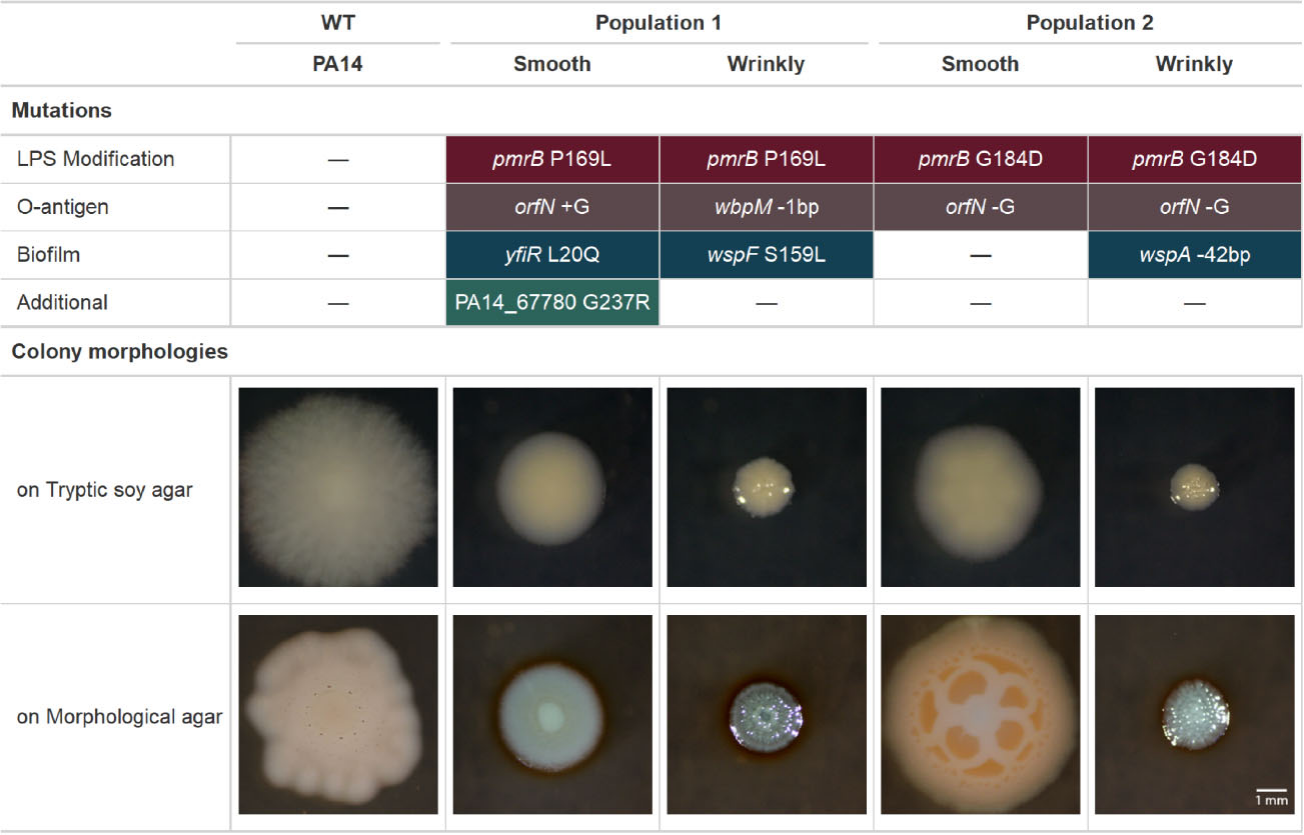
Isolated clones have at least two mutations involving LPS modification and O-antigen synthesis, some with additional biofilm mutations, affecting colony morphologies. Each wrinkly clone has a specific mutation that was not detected at the population level (*wbpM* and *wspA*), but they still fall into the functional categories associated with resistance shown in Fig. 3. The two wrinkly colonies have expected *wsp* mutations, while the two smooth morphotypes differ from their wild-type ancestor in size, shape and coloration. It is notable that three of four isolates acquired biofilm-associated mutations despite no explicit biofilm selection

**Figure 5.**
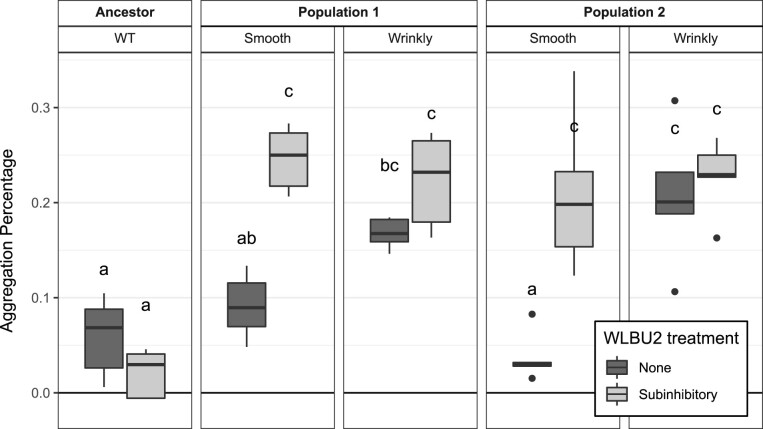
Aggregation of PA14 and resistant clones. The resistant Wrinkly (Wr) clones (associated with *wsp* mutations) aggregate more than WT regardless of WLBU2 treatment. The Smooth (Sm) clones did not differ from WT in the absence of WLBU2, but aggregated when exposed to WBLU2. Aggregation was measured as settling for 24 h at 4°C, *n* = 5. Means sharing a letter are not significantly different (Sidak-adjusted least-square means, *P* < 0.05). Dark and light gray denote culturing in the absence and presence of WLBU2, respectively

We propagated five planktonic populations of WT PA14 (MIC to WLBU2 = 4 ± 0 mg/l, Fig. 2) for 10 days under subinhibitory concentrations of WLBU2, followed by 2 days at inhibitory concentrations (Fig. 1). We estimated that the probability that any given nucleotide would be mutated during this regime is 0.74, with mean mutations per site of 1.52 (Table 1). Again, as a control, three populations were propagated in the absence of WLBU2 to distinguish between mutations adaptive to broth or WLBU2. Only two populations survived the prolonged subinhibitory treatment. Those two populations showed 3–4-fold increased resistance to WLBU2 and 2–3-fold increases to polymyxin B (Fig. 2). We also detected different colony morphologies within each population at the end of the experiment. Population 1 included small, rugose or wrinkly (Wr) colonies in addition to the smooth (Sm) morphology of the ancestor, and Population 2 contained both Sm and Wr colonies (Fig. 4). It is interesting to note that small colony variants are associated with aggregation, increased biofilm formation and worse outcomes in chronic infections [34, 35]. We tested whether these clones produced more biofilm by measuring aggregation in the presence or absence of WLBU2. We found that the Wr clones settled in clumps more than the ancestral strain both in absence and presence of WLBU2, while Sm colonies only clustered more in the presence of the peptide (Fig. 5).

WGS of the two surviving populations and representative clones of each colony type revealed that all acquired mutations in *pmrB* as well as mutations known to increase biofilm production (Fig. 3). The two small colony variants acquired mutations in *wspA* or *wspF* in the Wsp pathway seen previously in the hypermutator experiment, while one resistant Sm clone acquired *orfN* and *pmrB* mutations as well as *yfiR*, another gene known to activate c-di-GMP synthesis [36]. It is notable that these biofilm-activating mutations were selected during planktonic propagation of the populations, suggesting that forming aggregates is a key step for resistance to WLBU2. In addition, both resistant populations and the two clones with smooth morphology had mutations in the *orfN* gene, which is involved in the synthesis of the O-antigen [37]. Furthermore, one Wr isolate had a mutation in *wbpM*, which also contributes to O-antigen biosynthesis [37].

## DISCUSSION

The rapid emergence and dissemination of encoded resistance mechanisms is decreasing the effectiveness of antibiotics in the clinic. Antimicrobial peptides (AMPs) are components of the innate immune systems of many animals and plants and have been proposed as good candidates for treating multidrug resistant infections where other antibiotics have failed [4, 38]. AMPs derived from natural defensive peptides have advantages compared to traditional antibiotics, thanks to their broad-spectrum activity, ability to treat biofilm-embedded populations and observed evolutionary constraints that limit the rapid acquisition of AMP resistance [38–40]. In fact, numerous AMPs, including daptomycin, vancomycin and bacitracin, have been approved by the FDA, and several other AMPs, including WLBU2, are under clinical trials [41]. An important aspect of AMPs is that bacteria evolve resistance to them to a slower rate than to conventional antibiotics. This is due to several reasons, including their rapid bactericidal action, multiple targets in the bacteria including the cytoplasmatic membrane, and the frequent requirement of multiple mutations to produce resistance [1, 3, 40, 42, 43]. However, AMPs face some handicaps that have limited their successful development for clinical use, including low activity in acidic conditions such as blood or plasma, host toxicity, susceptibility to protease digestion and poor absorption [39]. Nevertheless, recent research with several engineered AMPs have demonstrated that most of these limitations can be overcome by structural re-design [39, 44].

WLBU2 is an engineered AMP derived from the natural LL-37 peptide that has overcome most of the limitations described above (reviewed in ref. [39]). The effectiveness of WLBU2 against a range of bacterial species—including the ESKAPE pathogens—in multiple conditions and the absence of clear genetic mechanisms for resistance makes it a promising candidate as a new antibiotic. However, one exception is its relative ineffectiveness against the *Burkholderia cepacia* complex [4], which is also generally resistant to the cationic peptide polymyxin B. Despite this exception, the lack of any defined resistance mechanism suggests that multiple modifications to LPS or outer envelope (e.g., associated with major species differences rather than variation at a single locus) may be required for WLBU2 resistance. Our study indicates that multiple mutations are indeed necessary for WLBU2 resistance in two of six ESKAPE pathogens. While further investigation is needed to determine if multiple mutations are needed to produce resistance in other pathogens, previous research has shown that the evolution of resistance to WLBU2 is slower than to colistin in several Gram-positive and Gram-negative pathogens, suggesting that multiple mutations will be required as well in other microbes [4]. This need for acquiring multiple mutations should substantially lower the probability of evolved resistance during treatment. For example, if a population of *P. aeruginosa* undergoes fewer than 10^9^ total cell divisions, double mutants in the resistance genes we identified are expected to occur in only 3% of treated infections (Table 1 and Supplementary Table S2). However, this pathway to resistance becomes much more likely for hypermutator strains or ones already containing one or more contributing mutations. Either possibility has been demonstrated in *P. aeruginosa* infections: hypermutator isolates are frequently reported [24], and mutations in the primary driver gene *pmrB*, as well as those in the Wsp pathway or affecting O-lipid biosynthesis are also common adaptations in clinical isolates. These mutations can be selected for their advantages in biofilms even in the absence of antibiotics [35, 45].

By exposing bacterial populations to antibiotics in controlled environments, we can understand the routes that lead to resistance [20, 27]. When new mutations are found in parallel in independently propagated lines in different strains and under conditions (for instance biofilm or planktonic) and not in the controls, then those mutations are the cause of the new heritable resistant phenotype. After sequencing all populations that survived the WLBU2 treatments (five using the hypermutator lineages, and two propagated under prolonged subinhibitory selection), the only mutations common across lineages occurred in *pmrB*, *orfN*, *morA* and *wsp* (Supplementary Table S5), providing clear evidence of fitness benefits of these mutations affecting both LPS composition and biofilm production in the presence of WLBU2 and their implication in the increase of resistance to WLBU2.

It has been well demonstrated that resistance to cationic peptides in *P. aeruginosa* involves modification of LPS [46]. Consistent with these findings, we detected parallel evolution of mutations in the *pmrB* and *orfN* genes across four independent lineages. PmrB forms part of a two-component regulatory system that modifies the lipid-A composition including its negative charge, a mechanism that is commonly associated with cationic peptide resistance in several species [29, 45]. In combination with *pmrB* variants, mutations in *orfN* and *wbpM* were found in WBLU2-resistant populations and clones. OrfN and WbpM form part of the operon that synthetizes the LPS O-antigen, and mutations in *orfN* are predicted to increase antibiotic resistance by reducing membrane permeability [20, 37]. Furthermore, we observed mutations in *wspA*, *wspF*, and *yfiR* that increase aggregation and/or biofilm production, likely through increased cyclic-di-GMP that in turn increases production of the cationic Pel polysaccharide. It will be important to explore how this positively charged component of the biofilm matrix interacts with the positively charged WLBU2 peptide to provide defense. Mutations in the phosphodiesterase domain of *morA* also were repeatedly selected in hypermutator populations with a probable similar effect on cyclic-di-GMP and Pel-mediated biofilm production [47]. Aggregation by secretion of a biofilm polymer is therefore another likely mechanism of WLBU2 resistance or tolerance [35, 48].

As AMR spreads around the globe, it is crucial to develop new antibiotics so that we still have treatments available for otherwise routine infections. During antibiotic development, we must anticipate possible mechanisms of resistance in their design [9, 10]. Furthermore, to broaden use of AMPs in clinical settings, we must explore evolutionary, genetic and phenotypic barriers to AMP resistance in relevant pathogens [40]. We often read claims of new ‘evolution-proof’ antibiotics [49], but here, we further demonstrate that the absence of resistance mechanisms can result from insufficient sampling of genetic diversity [50]. We also reveal the genetic causes of evolved resistance to the promising new cationic peptide WLBU2 by combining experimental evolution under multiple population-genetic conditions with genome sequencing of whole populations and resistant clones. Our findings indicate that WLBU2 likely has the advantageous property of requiring two or more mutations that affect the charge of the outer membrane as well as cellular aggregation for resistance to evolve. Further biochemical assays are needed to determine the discrete roles of each mutation in the resistance phenotype. This additional research notwithstanding, we believe that effort should be spent elucidating the evolutionary pathways driving antibiotic resistance when developing antibiotics, because knowing the likely adaptations could help in designing more accurate treatments, limit the spread of AMR and preserve the longevity of the antibiotic in clinical use.

## SUPPLEMENTARY DATA

Supplementary data is available at *EMPH* online.

## Supplementary Material

eoac022_Supplementary_Data
