# Supplementary material for: Evolved resistance to a novel cationic peptide antibiotic requires high mutation supply
Source: Evol Med Public Health. 2022 May 30;10(1):266–76. doi: 10.1093/emph/eoac022 (PMC9198447; doi:10.1093/emph/eoac022)
Supplement: eoac022_Supplementary_Data [file eoac022_supplementary_data.zip › eoac022_Supplementary_Data/Figure S1.docx]

Figure S1. Schematic representation of the biofilm propagation (top) and the planktonic propagation (bottom) used to propagate the sensitive strains in presence of WLBU2.

For biofilm populations (top), we transferred a polystyrene bead of the 24-hour culture to fresh media containing three sterile beads, which selects for adherent cells more than planktonic cells. Each day we alternated between black and white marked beads, ensuring that the bacteria were growing on the bead for 24 hr, which corresponds to approximately 6 to 7.5 generations/day. For the planktonic propagation (bottom), we serially passaged 50 µl into 5 ml of M9+ (dilution factor 100) which corresponds to approximately 6.67 generations per day.
