# Supplementary material for: Evolved resistance to a novel cationic peptide antibiotic requires high mutation supply
Source: Evol Med Public Health. 2022 May 30;10(1):266–76. doi: 10.1093/emph/eoac022 (PMC9198447; doi:10.1093/emph/eoac022)
Supplement: eoac022_Supplementary_Data [file eoac022_supplementary_data.zip › eoac022_Supplementary_Data/Supplementary Table Legends.docx]

**Supplementary Tables**

**Table S1.** Mutations selected in the presence of increasing concentrations of polymyxin B. Two sheets can be found in the Table S1: “PolymyxinB_raw” which includes all mutations called by breseq and “PolymyxinB_relevant” which include the mutations selected for in presence of polymyxin B but not selected in the absence of the antibiotic.

**Table S2.** Estimated mutation probabilities during experimental evolution.

**Table S3.** Mutations selected using the *mutS* deficient strain. Three sheets can be found in the document: “raw_mutations” include all mutations called by breseq, “no_controls_>0.1” include the list of mutations subtracting from the raw list all mutations happening in the populations evolving without WLBU2 and with lower frequency than 0.1, and “parallel_genes” which include the three genes that are targeted in more than one resistant population and are not present in any control.

**Table S4.** Mutations selected in the subinhibitory experiment. Two sheets can be found in the document: “populations” and “clones” include all mutations called by breseq in the evolving populations and clones respectively. Note that the curated list of mutations, subtracting those found in controls without antibiotic and the ancestral strain, is listed in Figure 2.

**Table S5.** Instances of parallel evolution events across the experiments. Four sheets are automated filtering and grouping of the raw mutations called by breseq for both mutS-founded and extended subinhibitory experiments (with mutations common to the ancestor removed) to highlight instances of parallelism: “All_mutations_>0.1” is all site-specific mutations at 10% or greater in any population, “All_mutations_>0.1_bygene” is this same list grouped by gene with cumulative frequencies, “Parallel_grouped_by_site” lists all site-specific mutations called in more than 1 population, and “parallel_grouped_by_gene” lists genes targeted across multiple mutations. “Grouped_bySite_Cleaned” lists the site-specific mutations seen in multiple populations and those seen at greater than 50% in the extended subinhibitory experiment, with erroneous mutation calls due to mismappings manually removed. “Grouped_byGene_Cleaned” further generalizes this curated list to the gene level with cumulative frequencies.
